# Effect of Saturation Pressure Difference on Metal–Silicide Nanopowder Formation in Thermal Plasma Fabrication

**DOI:** 10.3390/nano6030043

**Published:** 2016-03-07

**Authors:** Masaya Shigeta, Takayuki Watanabe

**Affiliations:** 1 Joining and Welding Research Institute, Osaka University, 11-1 Mihogaoka, Ibaraki, Osaka 567-0047, Japan; 2 Department of Chemical Engineering, Kyushu University, 744 Motooka, Nishi-ku, Fukuoka 819-0395, Japan; watanabe@chem-eng.kyushu-u.ac.jp

**Keywords:** nanopowder, metal silicide, co-condensation, thermal plasma, modelling

## Abstract

A computational investigation using a unique model and a solution algorithm was conducted, changing only the saturation pressure of one material artificially during nanopowder formation in thermal plasma fabrication, to highlight the effects of the saturation pressure difference between a metal and silicon. The model can not only express any profile of particle size–composition distribution for a metal–silicide nanopowder even with widely ranging sizes from sub-nanometers to a few hundred nanometers, but it can also simulate the entire growth process involving binary homogeneous nucleation, binary heterogeneous co-condensation, and coagulation among nanoparticles with different compositions. Greater differences in saturation pressures cause a greater time lag for co-condensation of two material vapors during the collective growth of the metal–silicide nanopowder. The greater time lag for co-condensation results in a wider range of composition of the mature nanopowder.

## 1. Introduction

Thermal plasmas have been used for effectual fabrication of nanopowders composed of nanometer-scale particles [1]. Nanopowders have unique capabilities that differ greatly from those of bulk materials or powders composed of larger particles [2]. Particularly, nanopowders composed of metal–silicide nanoparticles are anticipated to be potentially useful materials for extremely small electronic and mechanical applications such as solar-controlled windows, electromagnetic shielding, and contact materials in microelectronics [3]. However, because those raw materials usually have high melting points or boiling points, high-rate fabrication of those nanopowders is almost impossible using conventional methods such as grinding techniques and liquid-phase preparation. Combustion processes are also unusable because they are accompanied by unfavorable production of contaminants attributable to the oxidation atmosphere and because their flames cannot reach sufficiently high temperatures to vaporize the raw materials. Thermal plasmas offer the distinct benefits of high enthalpy, high chemical reactivity, variable properties, and a high cooling rate, all of which suit high-rate fabrication of metal–silicide nanopowders [4]. Additionally, the temperature and flow fields are controllable using external electromagnetic fields [5,6,7].

Thermal plasma fabrication of metal–silicide nanopowders involves the vaporization of raw materials and the subsequent conversion of the binary material vapors into numerous nanoparticles by virtue of the high enthalpy and high cooling rate of thermal plasma. However, the nanopowder growth is tremendously complicated because the nanopowder grows in a few tens of milliseconds through simultaneous and collective processes of binary homogeneous nucleation, binary heterogeneous co-condensation, and coagulation among nanoparticles with different compositions. Therefore, observing the growth process directly during experimentation is impossible. Only the characteristics of the final products have been evaluated [8,9,10,11,12]. Therefore, the growth mechanism remains poorly understood.

Computational studies based on theoretical modelling can reveal the growth mechanism and can enable prediction of the profile of the nanopowder to be synthesized. However, because of computational resource limitations, molecular dynamics (MD) calculation cannot comprehensively treat the entire growth process from nucleation until a nanopowder completes its growth [13]. In place of MD calculation with a heavy computational load, models based on aerosol dynamics have been used to simulate the process of a collective nanopowder growth comprehensively. Nevertheless, most models are applicable only to unary systems [14,15,16,17,18,19,20,21].

Only a few aerosol-dynamics-based models have been developed for thermal plasma fabrication of nanopowders involving co-condensations of binary material vapors [22,23,24]. Those models adopted several oversimplifications to obtain simple numerical solutions including only mean values. For more accurate and detailed numerical analysis in the growth processes of binary material nanopowders, we developed a unique model and solution algorithm [25,26,27,28]. That model can not only express any profile of particle size–composition distribution (PSCD) of a nanopowder even with widely ranging sizes from sub-nanometers to a few hundred nanometers, but can also simulate the entire formation process involving binary homogeneous nucleation, binary heterogeneous co-condensation, and coagulation among nanoparticles with different compositions. Especially for nanopowder formation of metal–silicides (Mo–Si, Ti–Si, Co–Si) under thermal plasma conditions, the model produced numerical results that agreed with experiment results [26,28].

Those computational results showed that the difference in saturation pressures between a metal and silicon was a crucial factor that determined the time lag of co-condensation and consequently affected the range of the silicon content in the synthesized nanopowder. Actually, an experimental study also reported that the saturation pressure difference affected the nanoparticle composition [11]. The literature emphasized that the difference of the saturation pressure caused that of the nucleation temperature and the larger difference resulted in a larger composition range of metal–silicide nanoparticles. Following this experimental study, the effect was investigated computationally using a simpler model as well [29]. Although the model did not consider binary nucleation and coagulation, it also predicted that systems with large differences of saturation pressures tended to produce metal–silicide nanoparticles with a wide range of compositions due to a time-lag of condensations of two materials.

Even though those studies indicated the importance of the saturation pressure difference between a metal and silicon, the effect remained unclear because the formation processes of nanopowders were also affected by material properties other than the saturation pressure. Data from the actual material properties were used for each material in those computations [26,28,29]. In experiments, it is generally impossible to control only a saturation pressure by changing materials. Other properties are changed as well. Therefore, in this study, numerical experiments are performed by changing only the saturation pressure of one material artificially to highlight the effects of saturation pressure differences on the metal–silicide nanopowder formation using the model which can simulate collective formation through simultaneous processes of binary nucleation, binary co-condensation, and coagulation among nanoparticles with different compositions [26,28].

## 2. Computational Conditions and Strategy

**Figure 1:**
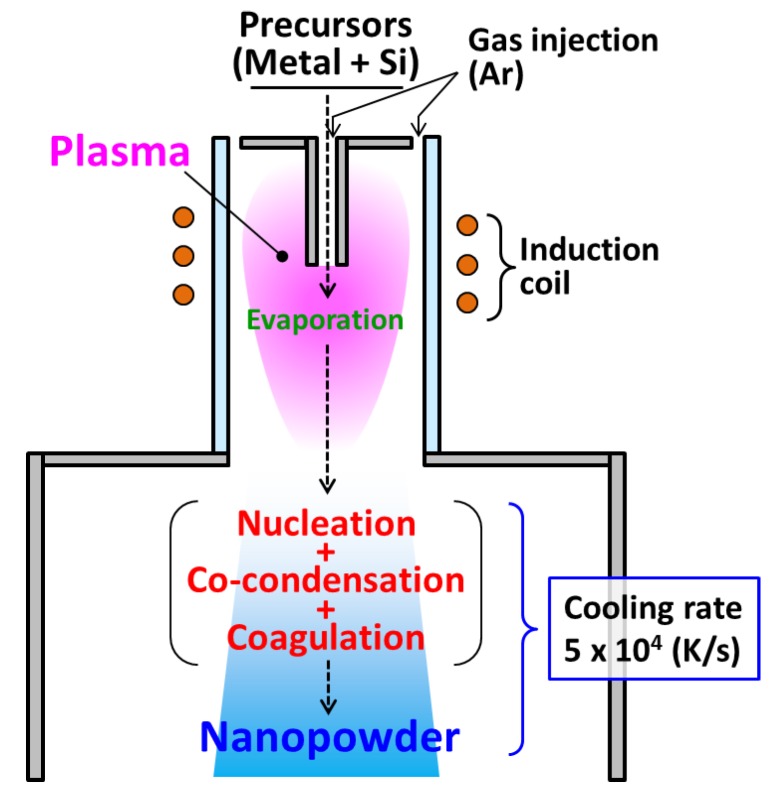
Metal–silicide nanopowder fabrication using an induction thermal plasma.

Figure 1 presents a schematic illustration of metal–silicide nanopowder fabrication using induction thermal plasma (ITP). The precursory raw materials are injected into a plasma where the high-temperature field vaporizes materials completely [22]. Metal and silicon vapors are transported with the flow to the plasma’s tail, which exhibits a rapid temperature decrease. Consequently, either or both of the material vapors become supersaturated, which engenders homogeneous nucleation. Because it is a binary system, nuclei composed of the metal atoms and silicon atoms are generated (binary nucleation). Immediately, the binary material vapors co-condense heterogeneously on the nuclei (binary co-condensation). Furthermore, during their growth, the nanoparticles mutually collide and merge into larger nanoparticles (coagulation). The metal–silicide nanopowder growth consists of these three processes that progress collectively and simultaneously. As a consequence, such nanopowders always have varieties of sizes and compositions, as shown in the experiments [11,22,28].

In a typical condition of ITP discharge, the region downstream from the plasma offers a high cooling rate of 10^4^–10^5^ K/s. Therefore, the present computation sets a constant cooling rate of 5.0 × 10^4^ K/s to investigate the effect of saturation pressure difference under extremely simple conditions. The initial mole fraction of the material vapor to argon gas is set to be 0.5%. This can be regarded as a dilute condition in which the effect of the raw material on the flow field is negligible. This study particularly selects a titanium–silicon binary system because the saturation pressures of titanium and silicon are mutually close (*p_S_*_(Ti)_/*p_S_*_(Si)_ = 10^−1^–10^0^). The initial ratio of these materials is set fairly at Ti:Si = 1:1. These conditions suggest that the vapors of titanium and silicon co-condense almost simultaneously.

As a great benefit of computational investigation, the value of only one saturation pressure can be changed strategically, which cannot be done in experiments. Therefore, this study defines an artificial saturation pressure *p_S_’* = ζ·*p_S_*. Controlling only the value of ζ from 10^−3^ to 10^3^, while using the other actual material properties [30] with no changes, the effect of saturation pressure difference is highlighted. Computations are performed with a time increment Δ*t* of 2.0 μs, which provides sufficient resolution for the present condition.

## 3. Outline of Metal–Silicide Nanopowder Formation Model

Formation of binary metal-silicide nanopowder from the vapor phase can be computed using a unique model developed by the authors [26,27,28]. The model with the PSCD describes a collective and simultaneous growth process of two-component nanoparticles in a binary vapor system through binary homogeneous nucleation, binary heterogeneous co-condensation, and coagulation among nanoparticles with different compositions. The PSCD is defined on a 2D coordinate system with two individual variables of the particle size and composition (here, silicon content), where nanoparticles composing a nanopowder are present only at the grid points [26].

In the model, the free energy of cluster formation *W* [31] is an important variable that dominates the nucleation and co-condensation:

(1) $$W=-n_{\left(A\right)}k_{B}T\ln\left(\frac{N_{mono(A)}}{N\prime_{S(A)}}\right)-n_{\left(B\right)}k_{B}T\ln\left(\frac{N_{mono(B)}}{N\prime_{S(B)}}\right)+\sigma \prime s\prime$$

where *N_mono_*_(*M*)_ stands for the monomer number density of material *M* (= *A* or *B*), *N’_S_*_(*M*)_ signifies the equilibrium monomer number density of material *M* in the saturated vapor over a bulk solution. In addition, σ*’* and *s’* respectively represent the surface tension and the surface area of the cluster. For binary clusters, σ*’* is estimated approximately as:

(2) $$\sigma \prime =\frac{n_{\left(A\right)}\sigma_{\left(A\right)}+n_{\left(B\right)}\sigma_{\left(B\right)}}{n_{\left(A\right)}+n_{\left(B\right)}}$$

Therein, *n*_(_*_M_*_)_ is monomers of material *M* contained in a binary cluster and σ_(*M*)_ is surface tension of material *M*. It is noteworthy that the nanoparticles are allowed to grow by condensation only when the free energy gradients for particle formation, *W*, is negative or zero. Therefore:

(3) $$\frac{\partial W}{\partial n_{\left(M\right)}}\leq 0$$

During a nanopowder growth process with a temperature decrease, the nanoparticles will be solidified. Then they can no longer increase their size as spherical particles by coagulation. In general, the solidification point depends on the material composition [32]. Furthermore, the solidification point decreases with the particle diameter [33]. Although these effects of the solidification point variation of binary-component nanoparticles were considered in our previous studies [26,27,28], the present study removes these effects to clarify only the effect of saturation pressure difference on the growth process.

## 4. Results and Discussion

**Figure 2:**
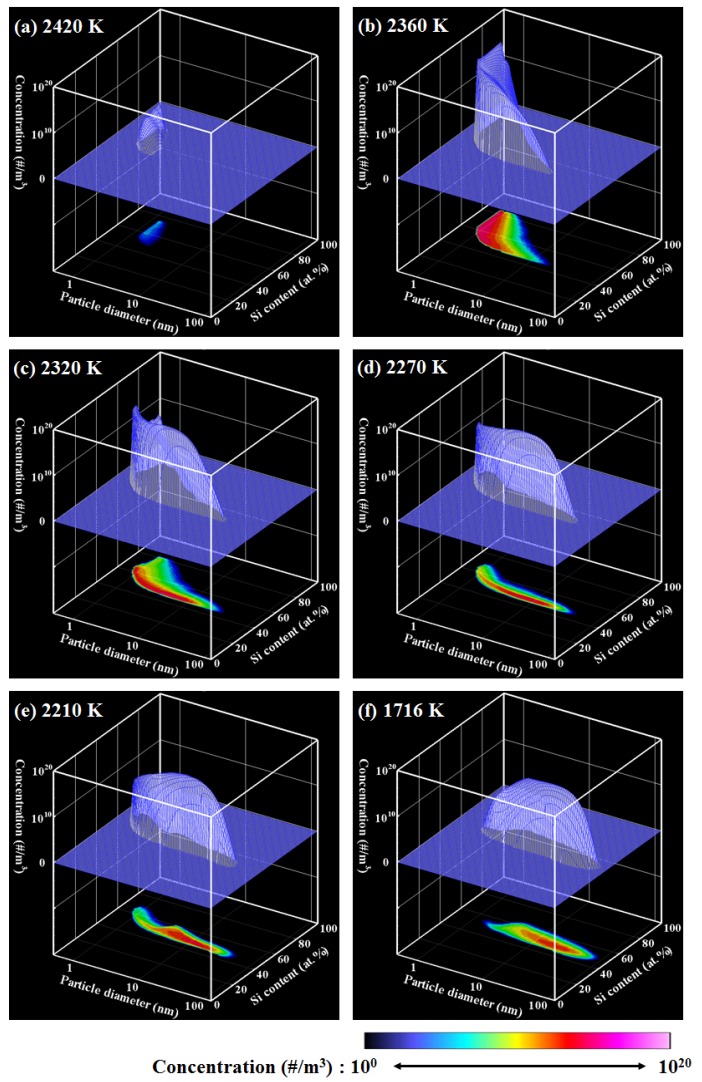
PSCD evolution for ζ = 1.

**Figure 3:**
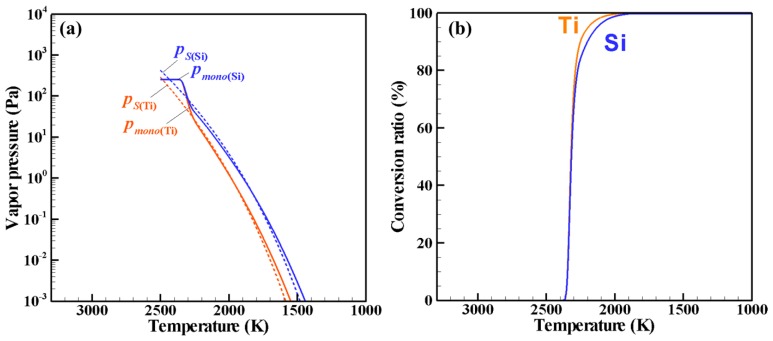
Phase conversion histories for ζ = 1: (**a**) vapor pressures and (**b**) conversion ratios.

Figure 2 shows the PSCD evolution of the Ti–Si nanopowder for ζ = 1, which describes the growth process in an actual Ti–Si binary system. Figure 3a presents the histories of the vapor pressures and the saturation pressures of Ti and Si, whereas Figure 3b depicts the conversion ratios that indicate how much of each material vapor has been converted into nanoparticles. It is noted that the horizontal axes show the temperature in the opposite direction because of the cooling process. According to an earlier study [34], the nucleation rate with 1 nucleus/cm^3^s can be conveniently observed experimentally. In this study, the nucleation is judged to start when the nucleation rate first exceeds this value. For the present case, nucleation starts when the vapors of Ti and Si are cooled to a temperature lower than 2444 K. Figure 2a shows that nuclei composed of Ti and Si are generated at the early stage of growth. At this time, the vapors of both Ti and Si are supersaturated, as shown in Figure 3a. Following nucleation, the nanoparticles grow rapidly (Figure 2b–e) by the simultaneous co-condensation of the material vapors on the nuclei as portrayed in Figure 3b as well as coagulation among themselves. Figure 3b also shows that 99% of Ti vapor completes the conversion at 2055 K, whereas 99% of Si vapor completes the conversion at 1977 K. After this drastic growth, the nanopowder grows slowly through coagulation and finally reaches its mature state of Figure 2f at 1716 K. To determine such a mature state of a nanopowder, the maximum difference of the particle number density at each node for Δ*t* was monitored. That monitored parameter was defined as:

(4) $$Q=\max\left(\frac{\left|{\hat{N}}_{i,j}^{\left(t\right)}-{\hat{N}}_{i,j}^{\left(t-\Delta t\right)}\right|}{{\hat{N}}_{i,j}^{\left(t-\Delta t\right)}}\right)$$

where

(5) $${\hat{N}}_{i,j}^{\left(t\right)}=\frac{N_{i,j}^{\left(t\right)}}{\rho_{g}^{\left(t\right)}}$$

and ρ*_g_*^(*t*)^ is the bulk gas density at the time *t*. When *Q* fell to less than 0.1, the nanopowder was determined to be mature. The mature nanopowder is composed mainly of the nanoparticles with the silicon content of *x*_(Si)_ = 50.0 atom %, which is identical to the initially given silicon fraction to the precursor. Particle diameters range widely from a few nanometers to 76 nm.

**Figure 4:**
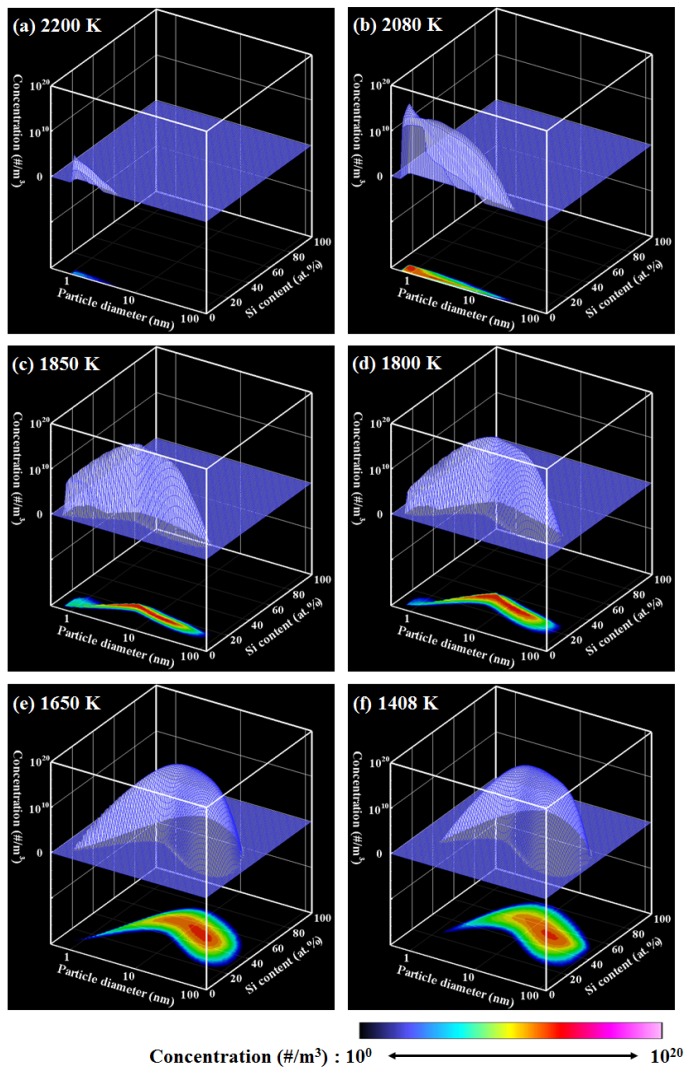
PSCD evolution for ζ = 10^3^.

**Figure 5:**
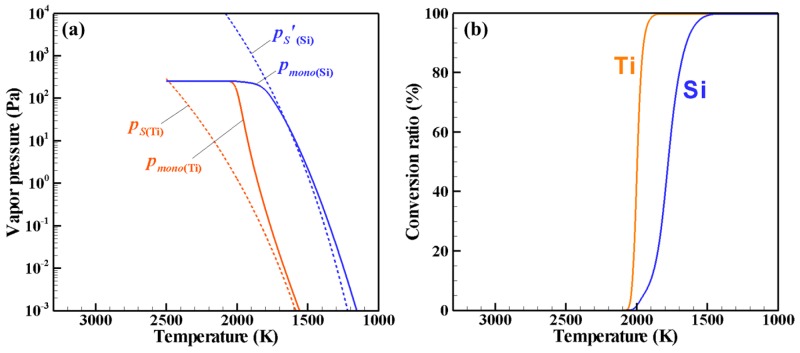
Phase conversion histories for ζ = 10^3^: (**a**) vapor pressures and (**b**) conversion ratios.

Figure 4 shows the PSCD evolution of the Ti–Si nanopowder for ζ = 10^3^. This value means that the saturation pressure of silicon is artificially set to be 1,000 times larger than the actual saturation pressure. Figure 5a,b respectively indicate the histories of the vapor pressures and the conversion ratios. Nucleation starts at 2258 K. At the early stage of growth, Ti-rich nuclei are generated (Figure 4a) and Ti-rich nanoparticles are formed (Figure 4b) only by Ti vapor condensation because the Si vapor pressure is still much lower than the saturation pressure (Figure 5a). During this growth of the Ti-rich nanoparticles, Si vapor starts to co-condense on the Ti-rich nanoparticles with Ti vapor (Figure 4c–e). Figure 5a shows that 99% of Ti vapor completes the conversion at 1882 K. After this co-condensation, Si vapor continues to condense on the Ti–Si nanoparticles slowly; 99% of Si vapor is consumed at 1506 K. The nanopowder reaches its mature state shown in Figure 4f at 1408 K. Although most the mature nanopowder exhibits silicon contents of *x*_(Si)_ = 50.0 atom %, the composition ranges widely from 25 atom % to 80 atom %.

**Figure 6:**
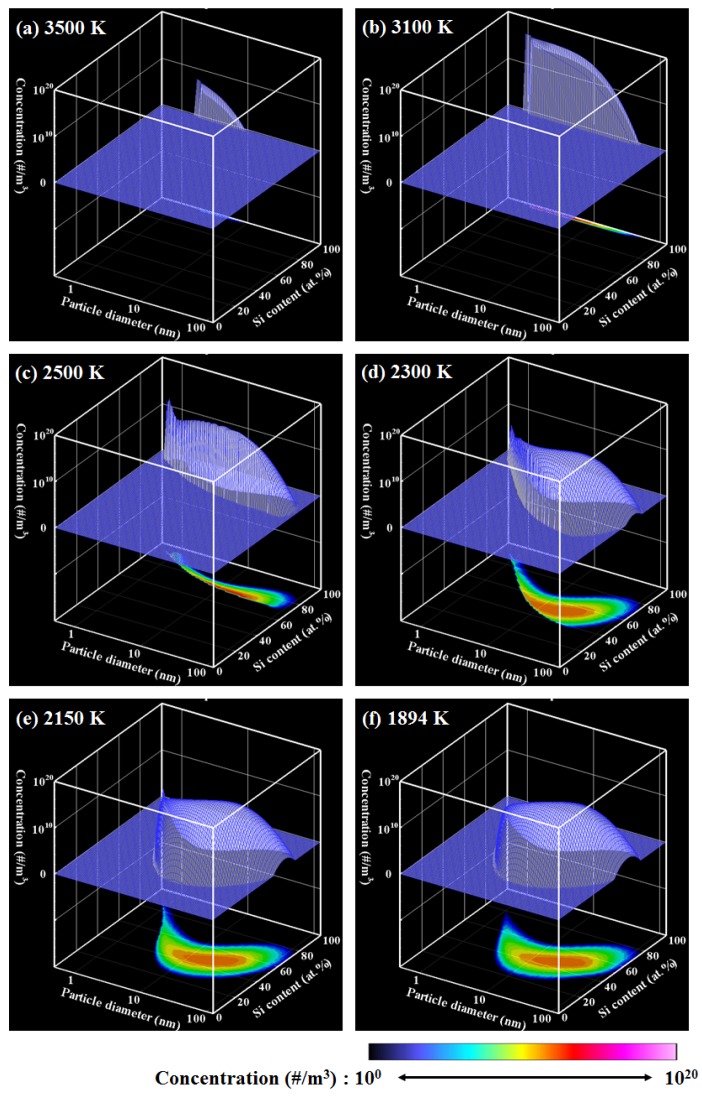
PSCD evolution for ζ = 10^−3^.

**Figure 7:**
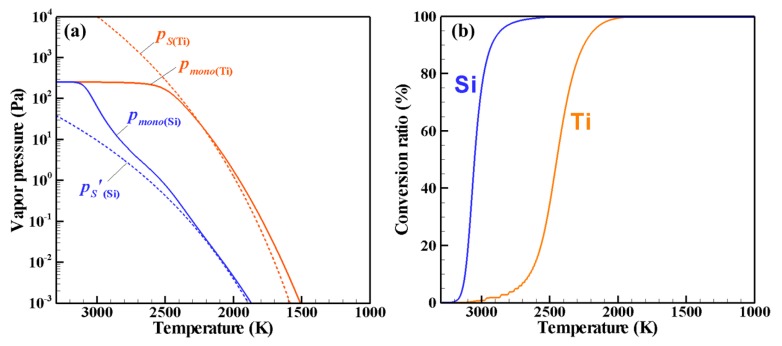
Phase conversion histories for ζ = 10^−3^: (**a**) vapor pressures and (**b**) conversion ratios.

Figure 6 shows the PSCD evolution of the Ti–Si nanopowder for ζ = 10^−3^. Figure 7a,b respectively depict the vapor pressures and the conversion ratios. Nucleation starts at 3777 K, which is much higher than in the other cases. At the early stage of the growth, Si-rich nuclei are generated (Figure 6a) and Si-rich nanoparticles are formed (Figure 6b) by Si vapor condensation because only the Si vapor pressure is supersaturated (Figure 7a). Following this growth of Si-rich nanoparticles, Ti vapor starts to co-condense on the Si-rich nanoparticles with Ti vapor (Figure 6c–e). 99% of Si vapor is consumed at 2654 K, at which only 9% of Ti vapor is consumed (Figure 7b). 99% of Ti vapor completes the conversion at 2031 K. The nanopowder reaches its mature state as seen in Figure 6f at 1894 K. This mature nanopowder also has widely ranging silicon contents, from 25 atom % to 80 atom %.

**Figure 8:**
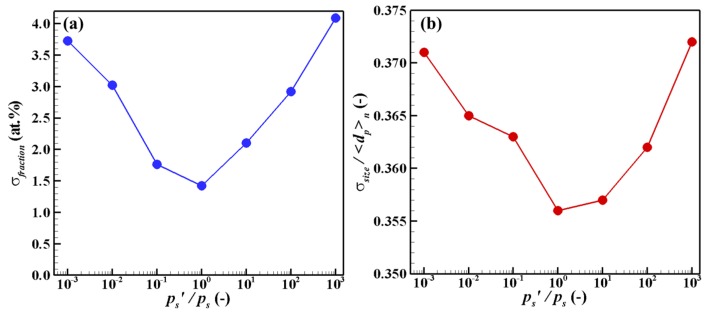
Effects of saturation pressure difference on dispersion of (**a**) fraction and (**b**) size.

These results imply that a wider range of composition is caused by a larger time lag of co-condensation that originally results from a larger difference of saturation pressures. Figure 8 shows the results obtained in the same manner using numerical experiments. As presumed, the standard deviations of fraction are larger when the saturation pressure differences are larger. Additionally, the results show that the standard deviations of size normalized by the arithmetic mean diameters are larger when the saturation pressure differences are larger.

## 5. Conclusions

Using our unique model and solution algorithm, which can simulate the entire growth process involving binary homogeneous nucleation, binary heterogeneous co-condensation, and coagulation among nanoparticles with different compositions, numerical experiments were carried out by changing only the saturation pressure of one material artificially to highlight the effect of the saturation pressure difference between a metal and silicon on the nanopowder formation in thermal plasma fabrication. A greater difference in saturation pressures causes a larger time lag of co-condensation of two material vapors during the collective growth of the metal–silicide nanopowder. The longer time lag of co-condensation results in a wider range of compositions for the mature nanopowder.
